# The scientific paper on clinician's perspective

**DOI:** 10.1590/2177-6709.21.5.015-016.edt

**Published:** 2016

**Authors:** David Normando

**Affiliations:** 1 Adjunct professor, Universidade Federal do Pará (UFPA), School of Dentistry, Belém, Pará, Brazil. Coordinator, Universidade Federal do Pará (UFPA), Graduate program in Dentistry, and ABO-Pará, Specialization course in Orthodontics, Belém, Pará, Brazil.

*"Every artist should go where the public is."* Milton Nascimento (Brazilian songwriter)

Oftentimes I question myself why does presentations in congresses arouse so much greater interest in clinicians than the reading of a paper, even if the topic of the publication is related to clinical aspects. There are many reasons. Conferences are interactive and doubts may be promptly answered. Images and videos may be inserted, causing perceptions unavailable in most of scientific publications. Besides, in presentations, results of any research may be complemented by clinical cases, making information visually enriched. It is fascinating. There are many other advantages about conferences presentations. And disadvantages too. 

Conferences are not auditable, although they can be immediately questioned. The information spread may have been manipulated and not represent the truth. Moreover, presentations based solely in clinical cases may represent information with lower level of evidence (proofs), implying in a higher risk for those patients undergoing the clinical procedure presented. Also, there is great chance of information biased by interest of the lecturers. The published science also has drawbacks in this regard, but it was previously scrutinized, questioned and revised before arriving to the eyes of the clinician and mouth of patients.

Conferences in events will not change. They are a huge success in the showcases of congresses around the world. As we observe striking changes in the layout of audiovisual presentations, the scientific papers repeat, for decades, the same *modus operandi*.

Couldn't they use a different layout? A more palatable one, with greater interactivity and a more objective language, but keeping its judicious template? I think we, editors and authors, should unite in this purpose and present our papers in a more dynamic and attractive layout, without losing sight of the accuracy inherent to the science. Many scientific journals of great impact are already restructuring the layout of their publications. In these journals, the manuscripts are more succinct and far more attractive, with a presentation combining images and videos. However, Dentistry and Orthodontics remain tied to the traditional layout.

Let us imagine a clinician reading a scientific paper. I believe most people start reading by the title, and then jump straight to the conclusion. Period. The main reason is the difficulty in understanding the whole content of the paper, specially the Methods. Moreover, the minority who tries to read through the Introduction will stumble at Materials and Methods. In my opinion, this part, "sandwiched" between Introduction and Results - information which the clinician really needs -, impairs the reading of the paper and threatens the interest of reading it thoroughly. 

One of the editor's role is to facilitate the interest for his periodical, making it more attractive for its readers. Science can not be edified only for those who create it, but mainly for clinicians who use the knowledge to treat their patients with more quality. Particularly, I think that the methods description could be placed in the end of the paper, and this will be only one of the many changes that the *Dental Press Journal of Orthodontics* will enplane from 2017. It comes strung to a greater motivation so that researchers publish videos about their studies and clinical cases related to the object of the investigation. The intention is the same one that Milton Nascimento poeticized in the lyrics of "Nos Bailes da Vida" (In the Dance of Life): To bring science closer to the clinician and bring more people to the dance.

David Normando - editor-in-chief (davidnormando@hotmail.com)

